# Supplementary figures and images for: Selective killing of homologous recombination-deficient cancer cell lines by inhibitors of the RPA:RAD52 protein-protein interaction
Source: PLoS One. 2021 Mar 30;16(3):e0248941. doi: 10.1371/journal.pone.0248941 (PMC8009417; doi:10.1371/journal.pone.0248941)

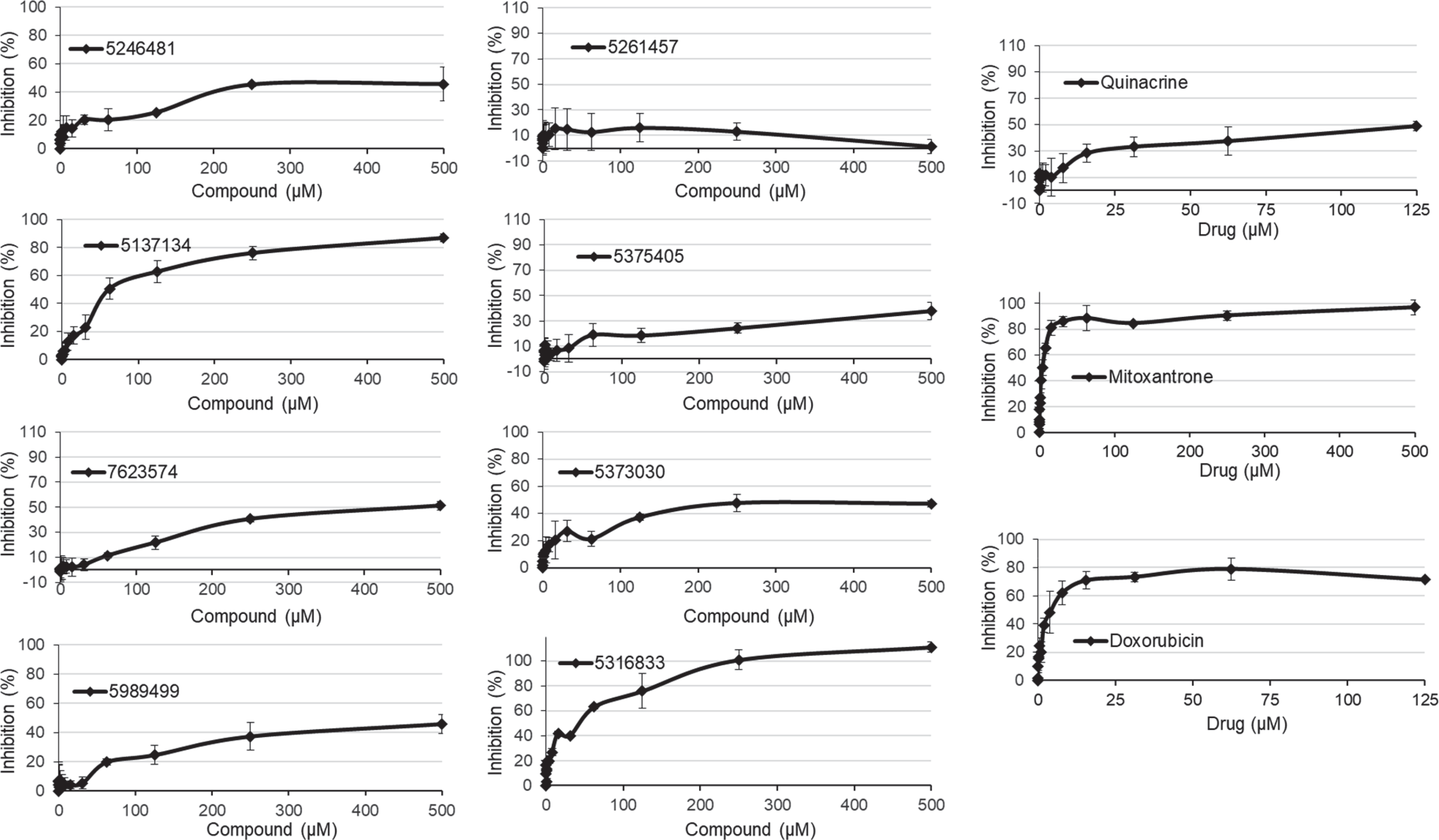

Supplement: S1 Fig — Sixteen treatment concentrations were used (0.030–500 μM) increasing by two-fold increments of each molecule/drug obtained as a hit in HTS FluorIA. RAD52(1–303) without GFP-tag and buffer (including DMSO) only were used as controls. Percent inhibition was calculated as follows: [(RFUSMI—RFURAD52(1–303)) / (RFUDMSO—RFURAD52(1–303))]*100, where RFU is relative fluorescence unit. An average of triplicate measurements was used. The treatment points for quinacrine and doxorubicin beyond 125 μM were showing characteristics of aggregation and were not used. (TIF) [file pone.0248941.s001.tif]

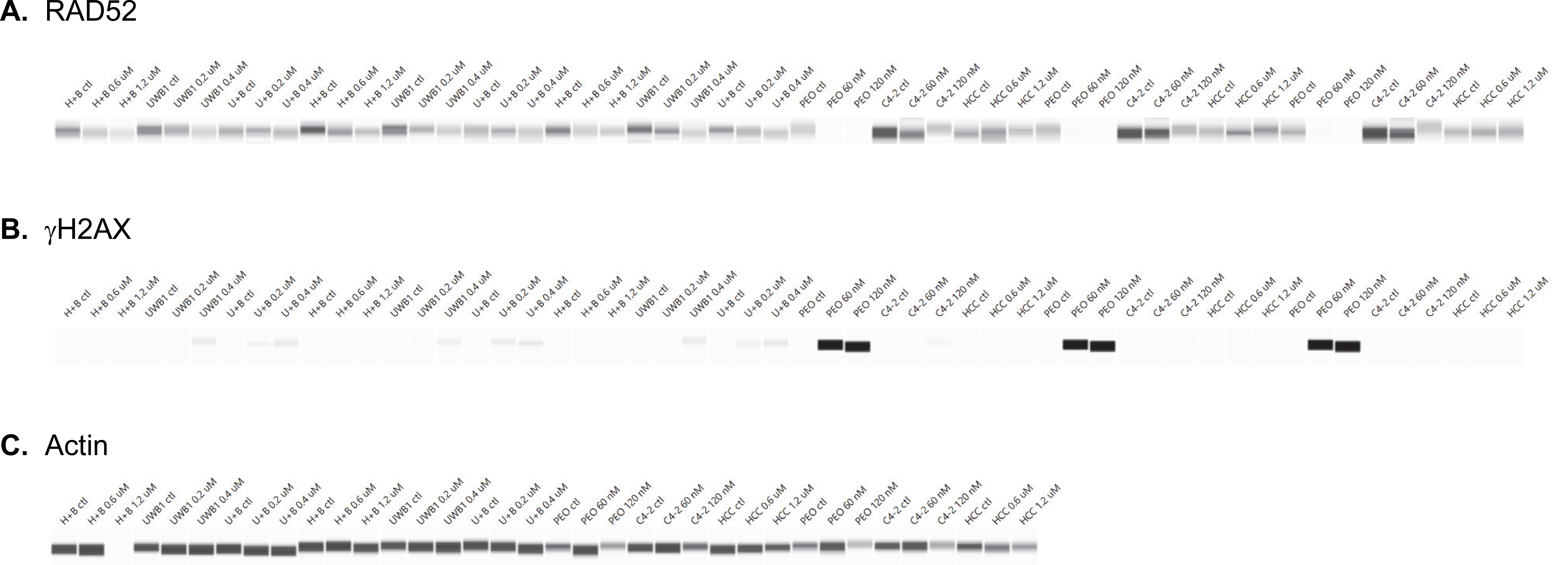

Supplement: S2 Fig — Data were collected on a nanocapillary PeggySue instrument and displayed with Compass software (Protein Simple) in “lane” mode. (A) Results from Abcam anti-RAD52 antibody (ab18263, diluted 1:25). Each sample was run three times. The RAD52 band at 56–58 kDa was confirmed using Phoenix cells (ATCC) overexpressing human RAD52 (not shown). (B) Results from R&D Systems Human Phospho-histone H2AX (S139) antibody (AF2288, diluted 1:100). Each sample was run three times and the band at 25 kDa is shown. (C) Results from Cell Signaling Technology Pan-Actin antibody (4968, diluted 1:2500). Each sample was run two times and the band at 48 kDa is shown. (TIF) [file pone.0248941.s002.tif]

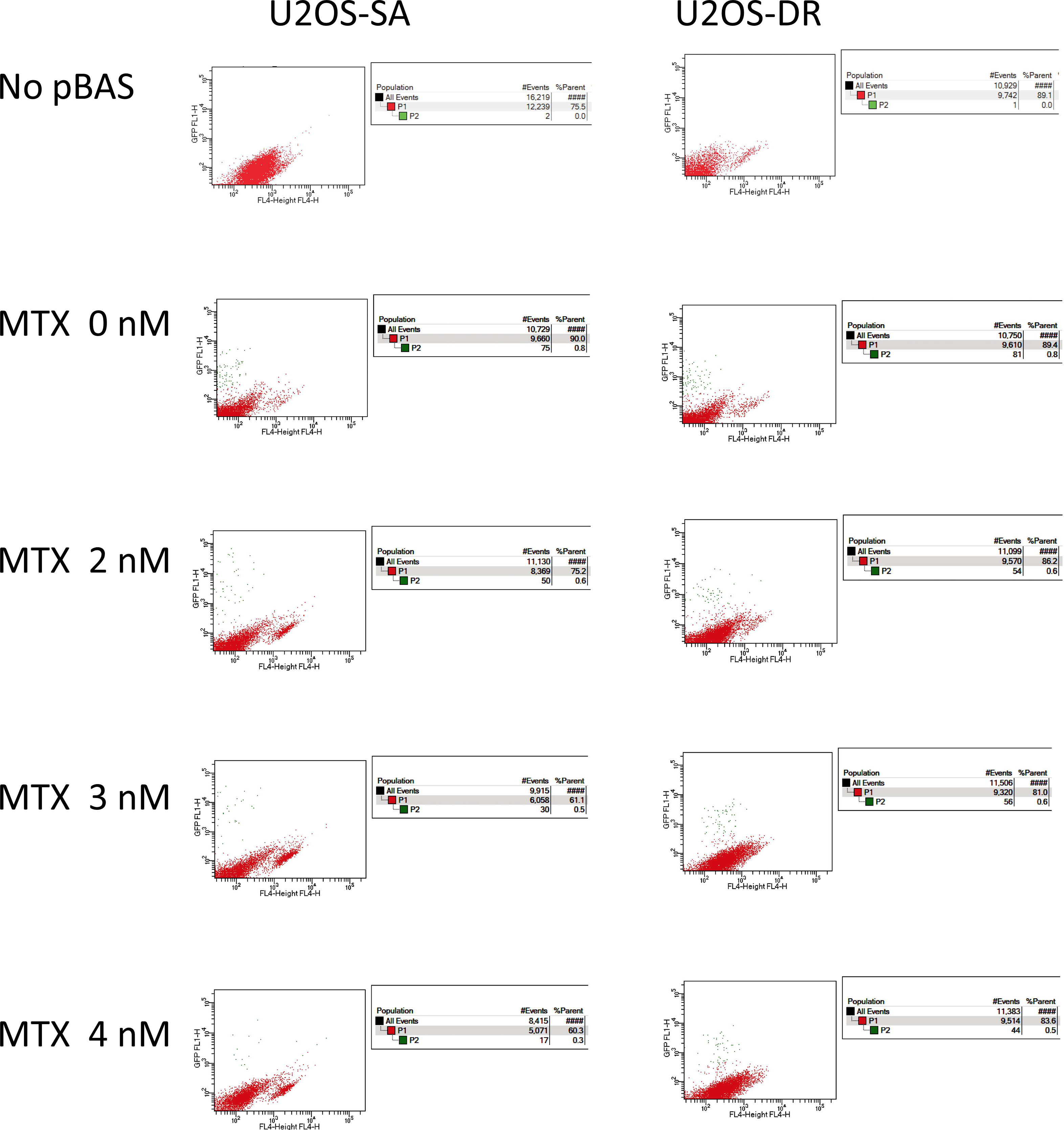

Supplement: S3 Fig — DSB repair activity by single strand annealing (SSA) or homologous recombination (HR) in MTX-treated GFP-reporter U2OS-SA and U2OS-DR respectively are measured by FACS analysis. Treated and untreated cells are sorted and repair activity by either pathway is measured by increased green fluorescence (y-axis). Green cells are calculated as a percent from total cells within each contour plot. (TIF) [file pone.0248941.s003.tif]

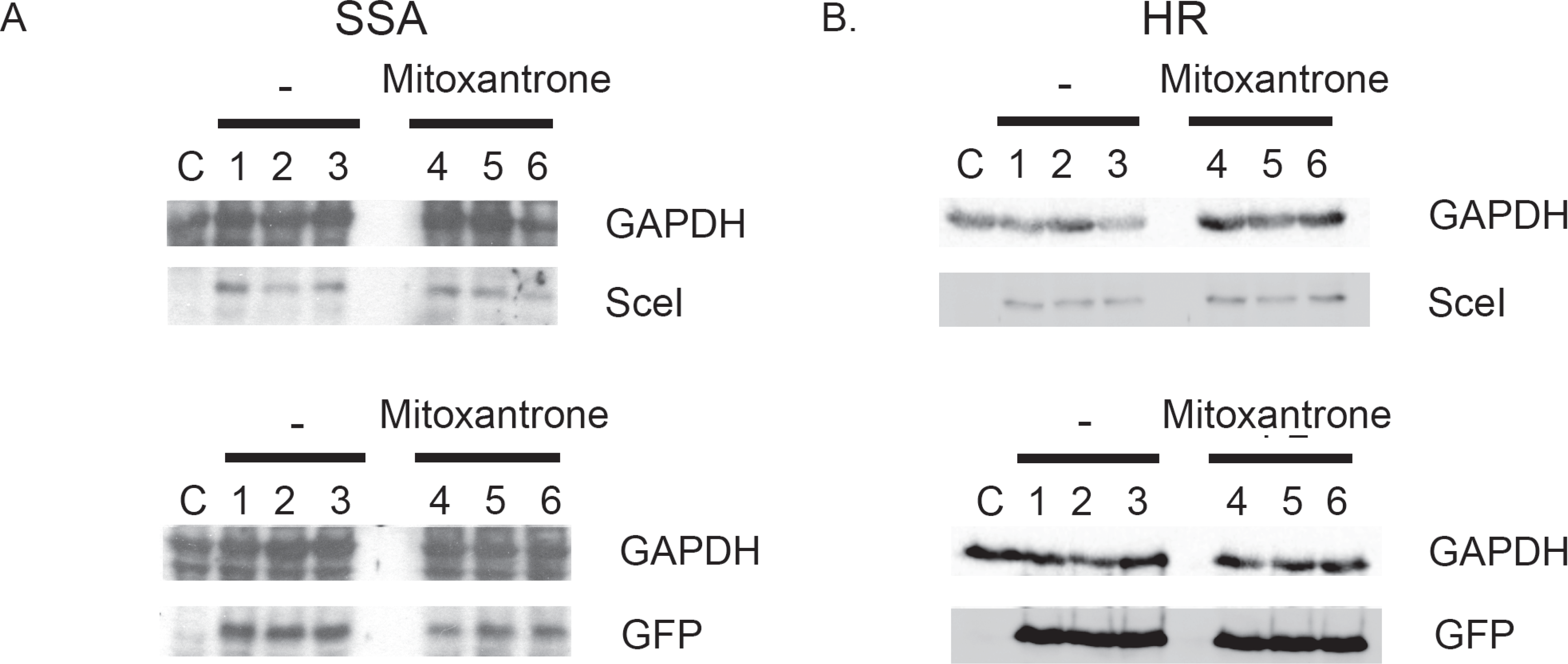

Supplement: S4 Fig — SSA (panel A) and HR (panel B) activity were determined by western blotting. The expression levels of I-SceI and GFP were determined by western blots. GADPH was used as a loading control. C: control experiments without I-SceI expression. Lanes 1–3; three independent experiments without mitoxantrone-treatment, lanes 4–6; three independent experiments with 3 nM mitoxantrone treatment. Cell lysate from each treatment was separated by 12% SDS-PAGE. Two identical samples were analyzed for one set of experiments, and one gel was used for I-SceI expression and the other was used for GFP expression. I-SceI and GFP signals were normalized by the signals of GAPDH in each lane. The repair activity in each lane was expressed as a ratio of normalized GFP/normalized I-SceI. (TIF) [file pone.0248941.s004.tif]

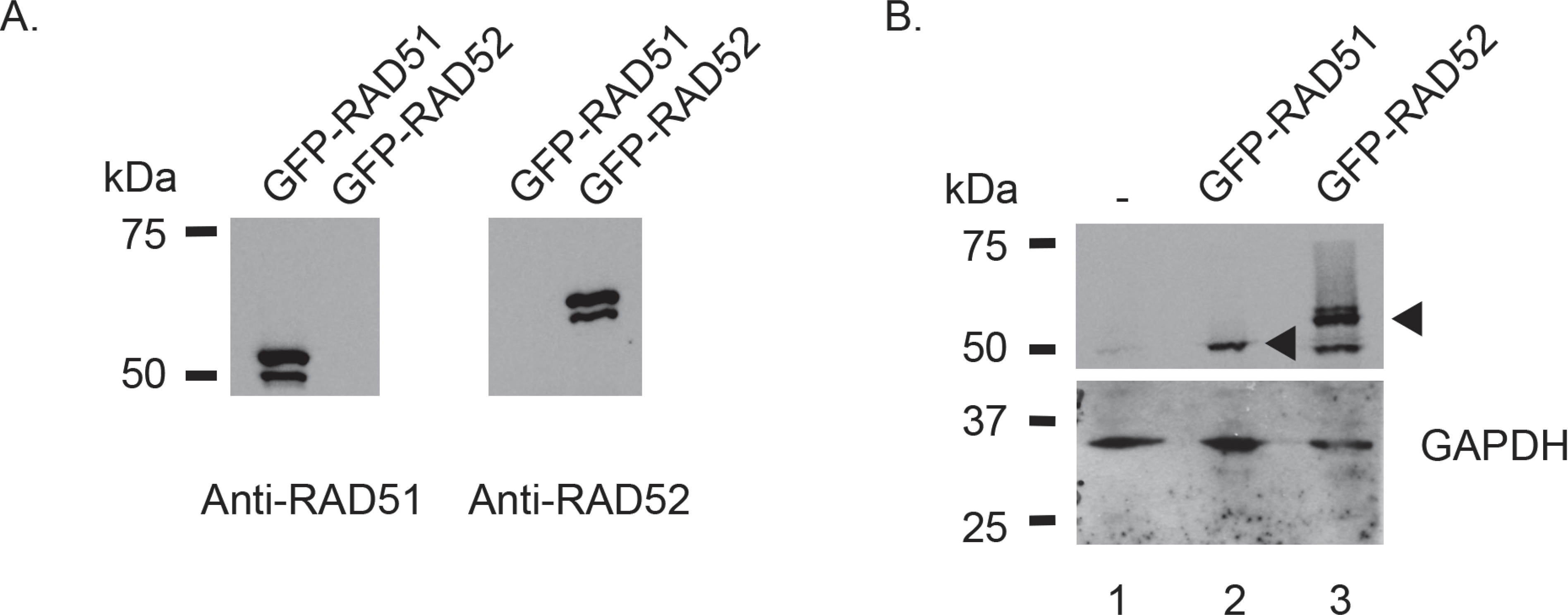

Supplement: S5 Fig — (A) GFP-RAD52 or GPF-RAD51 were immuno-precipitated by anti-GFP antibody (SCBT B-2), and the immuno-complexes were analyzed on 8% SDS-PAGE followed by the western blots with anti-RAD51 antibody (SCBT H92) and anti-RAD52 antibody (LSBio aa360-375). (B) Expression levels of GFP-RAD52 and GFP-RAD51. Cell lysates from control cells (lane 1), GFP-RAD51 expressing cells (lane 2), and GFP-RAD52 expressing cells (lane 3) were analyzed on 8% SDS-PAGE followed by western blots with anti-GFP antibody (GenScript pAb Rabbit). The arrows indicate GFP-RAD51 (lane 2) and GFP-RAD52 (lane 3). GAPDH was used as a loading control. (TIF) [file pone.0248941.s005.tif]
