# Supplementary material for: Selective killing of homologous recombination-deficient cancer cell lines by inhibitors of the RPA:RAD52 protein-protein interaction
Source: PLoS One. 2021 Mar 30;16(3):e0248941. doi: 10.1371/journal.pone.0248941 (PMC8009417; doi:10.1371/journal.pone.0248941)
Supplement: S1 Raw images — (PDF) [file pone.0248941.s006.pdf]

Figure 5

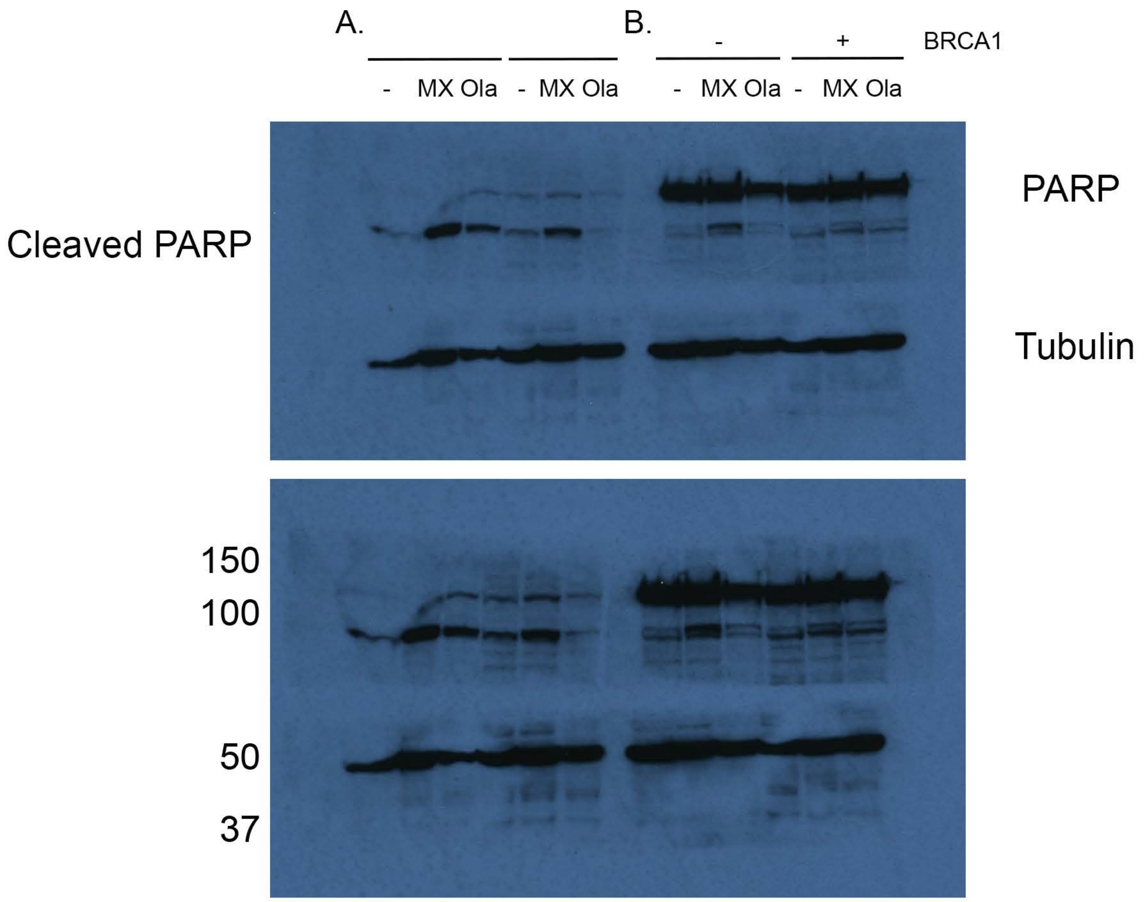

Images from two different exposure time to the film.
Images were obtained by scanning the film.

Figure 6 A. RAD52

+zeocin control

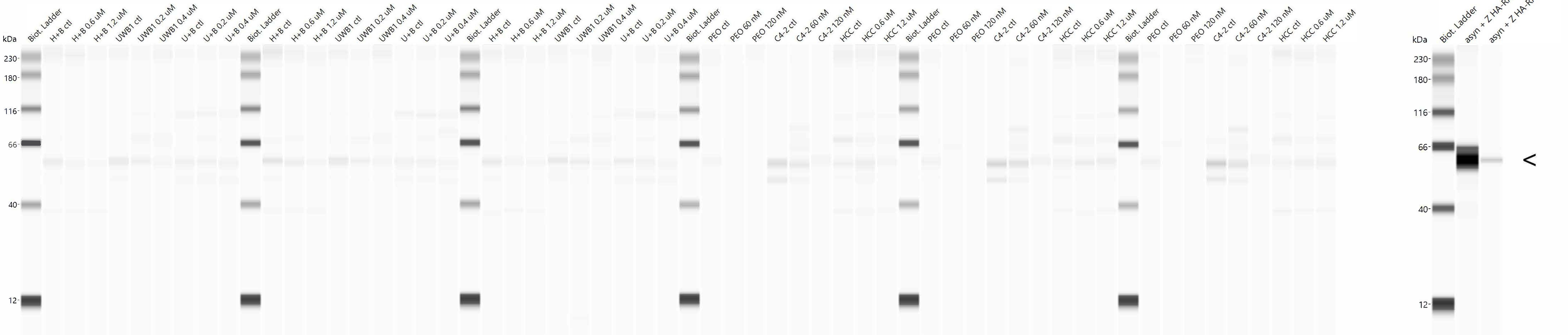

Figure 6 B. γH2AX

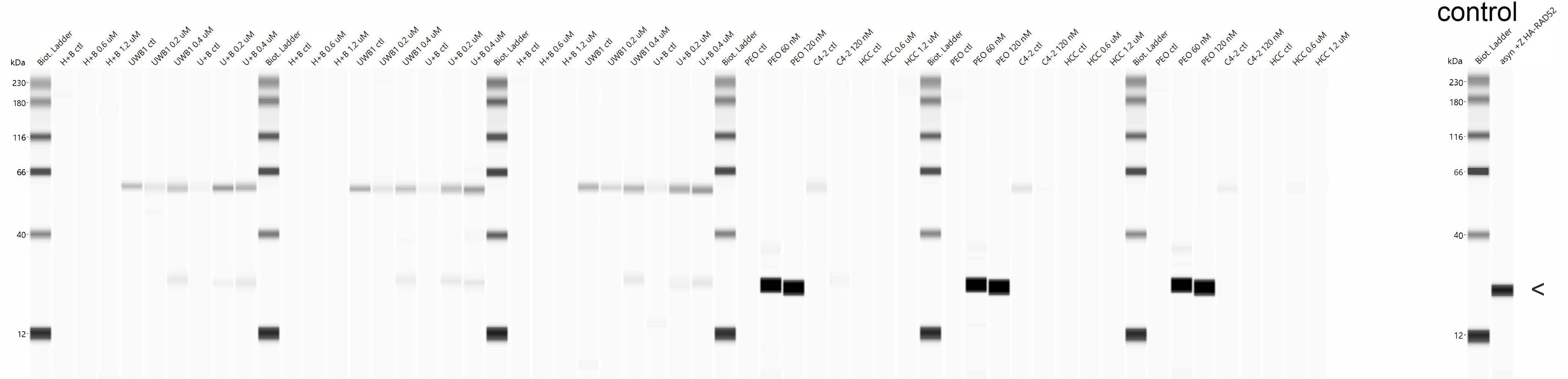

C. Actin

Figure 6

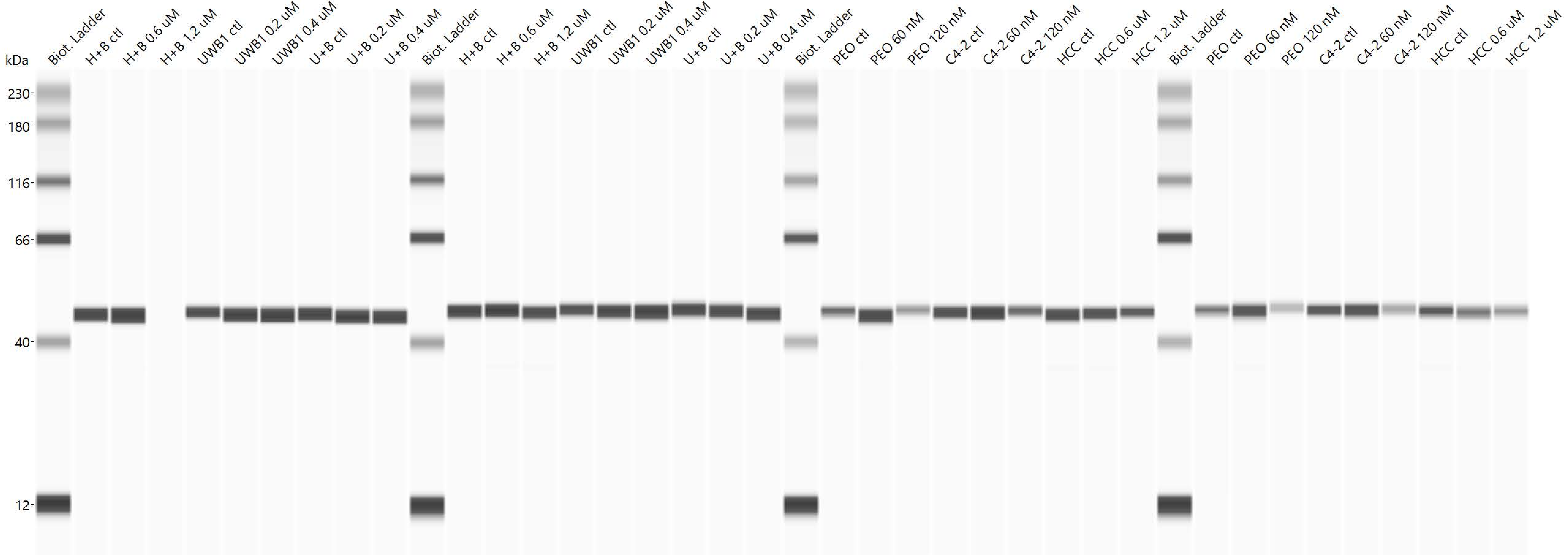

Figure 7C

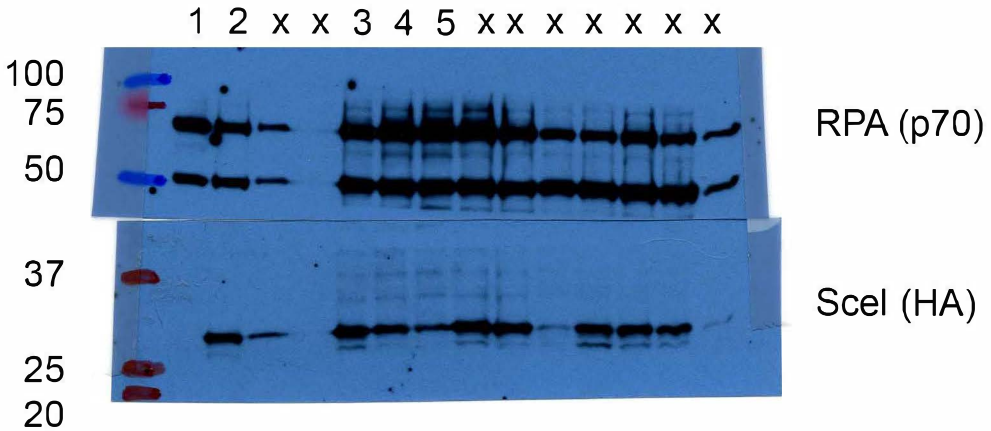

Lanes 1 to 5 were used to prepare the figure.

Image of X-ray film was obtained by scanning each film.

Figure 9

Control

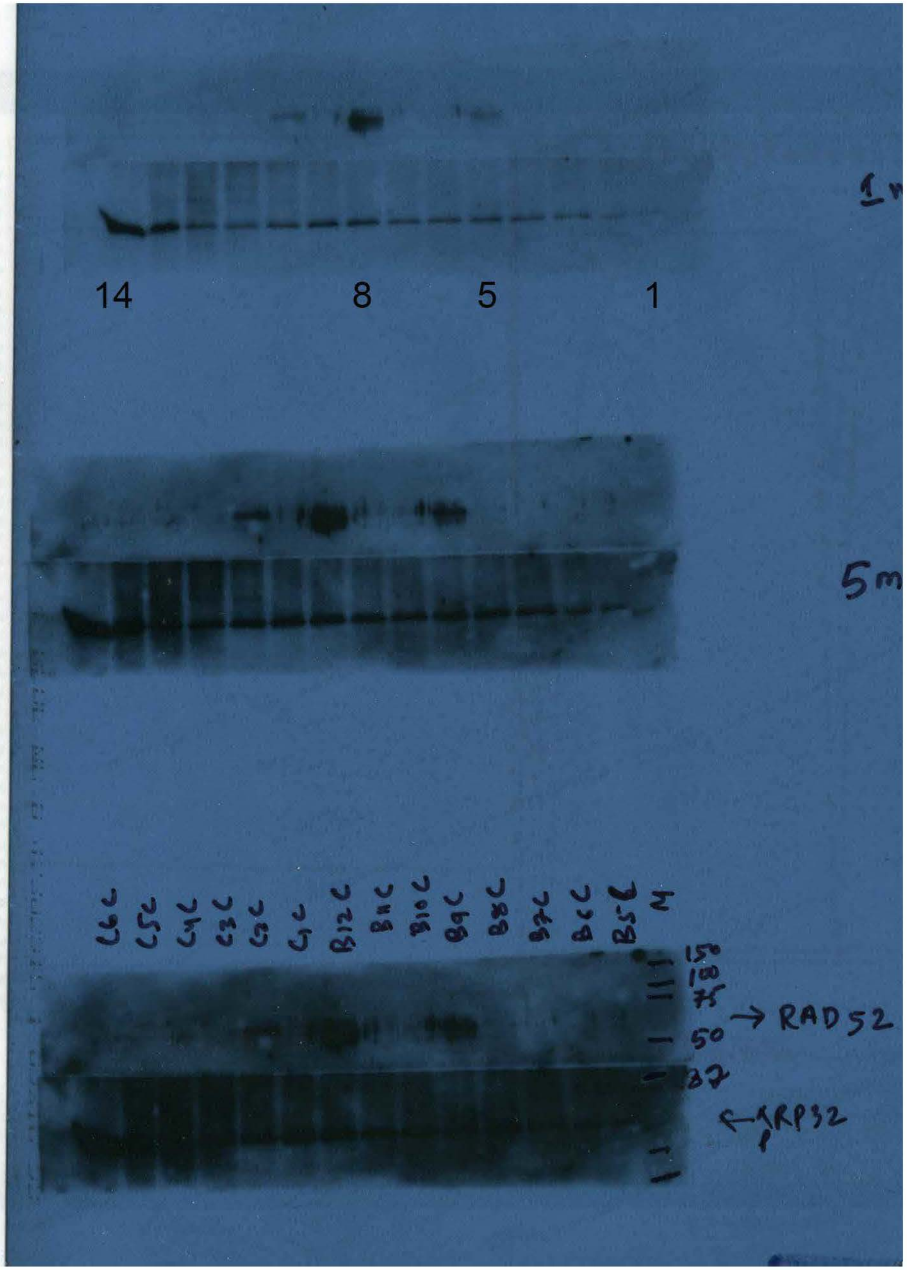

MTX at 3 nM

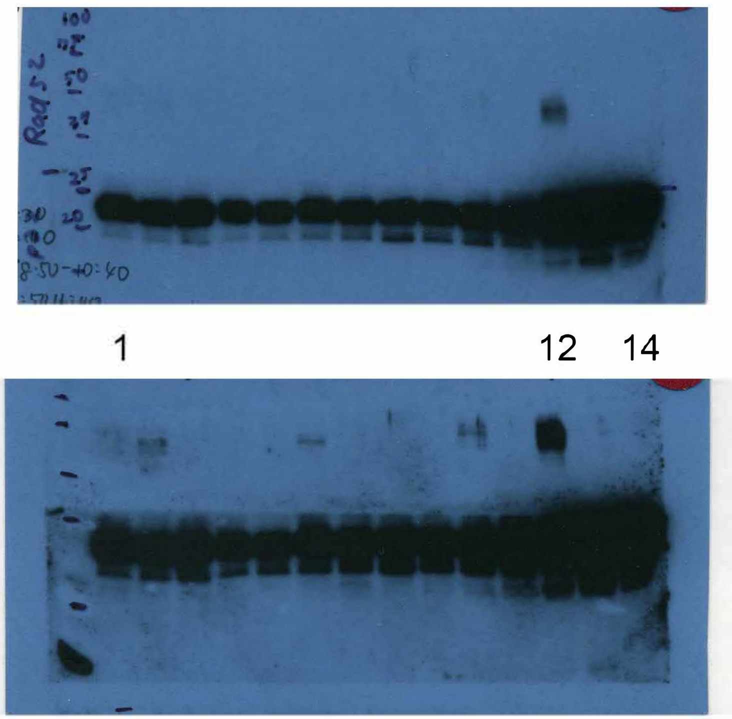

RAD52

RPA (p32)

RAD52

RPA (p32)

Control and treated with 3 nM MTX
two exposures are shown for each:
1 min (top) and 5 min (bottom)

Supplemental Figure 4

A. SSA

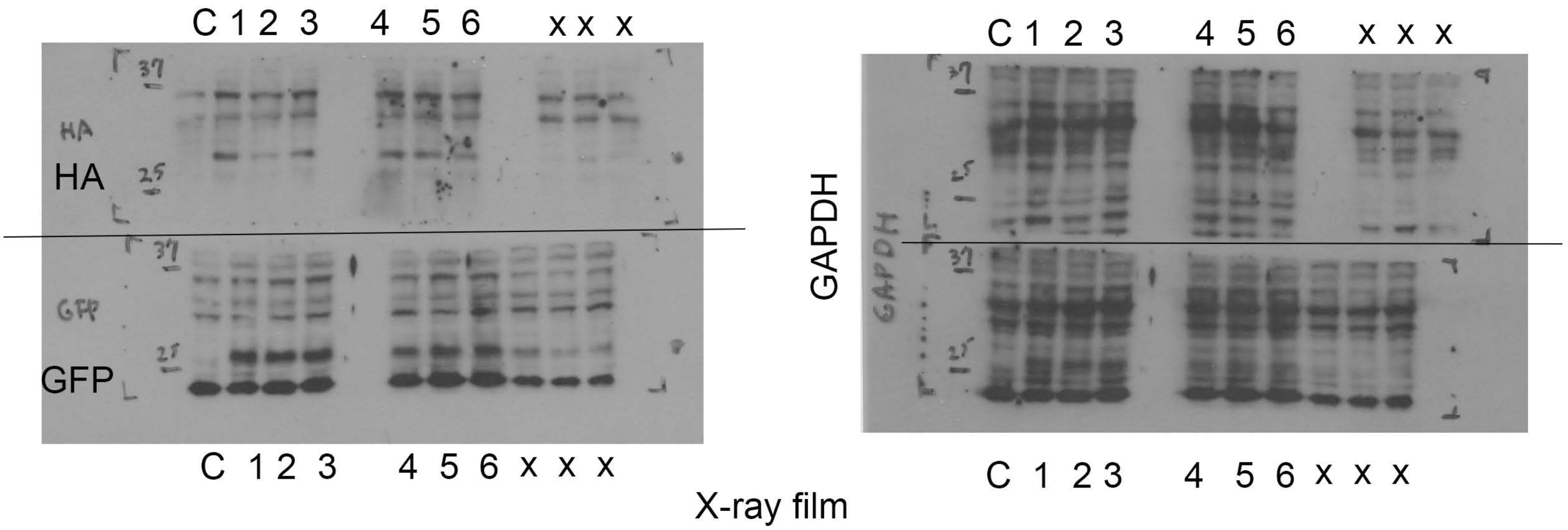

B. HR

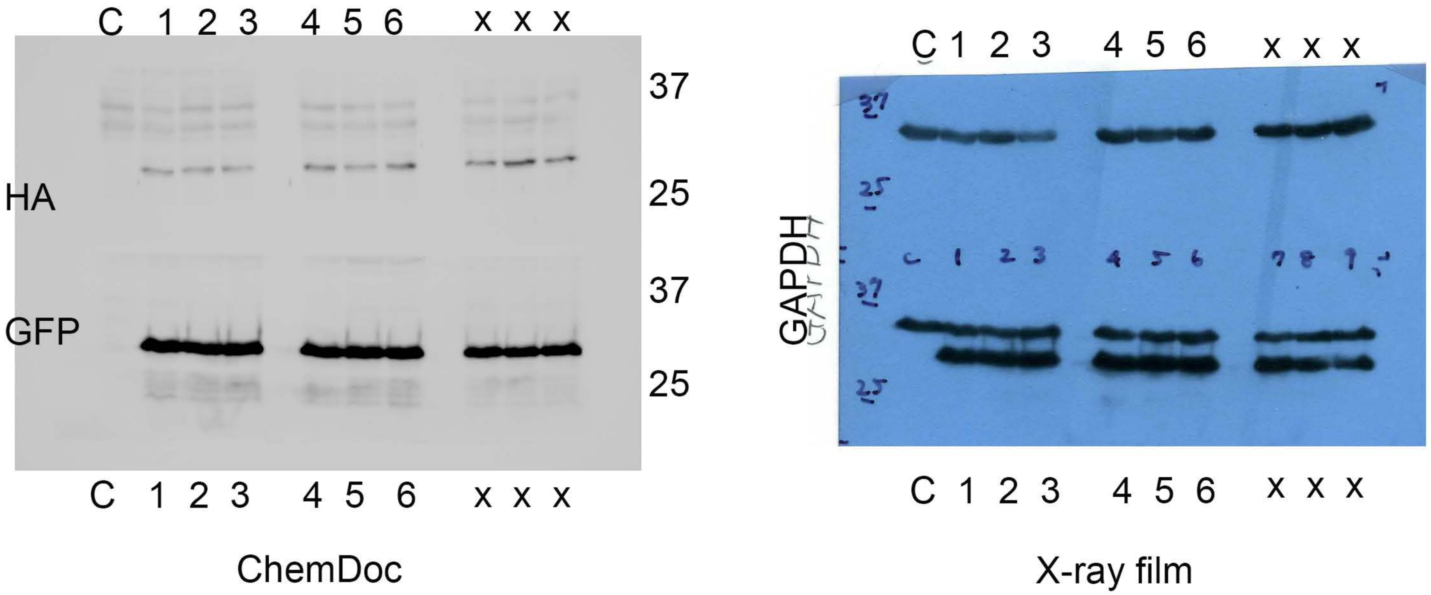

Image of X-ray film was obtained by scanning each film.

Supplemental Figure 5

A.

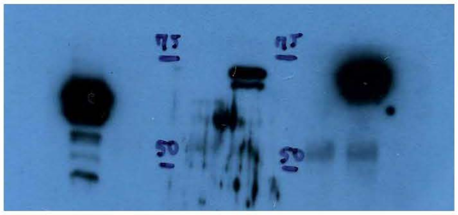

X-ray film

The same blot was exposed
to the film and scanned by ChemDoc.

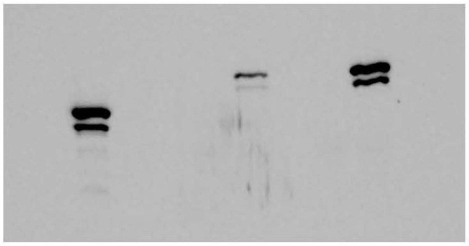

BioRad ChemDoc

1 2 x x 3 4

ChemDoc image was used.
1 and 3 : GFP-RAD51
2 and 4 : GFP-RAD52

Image of X-ray film was obtained by scanning each film.

B.

X-ray film

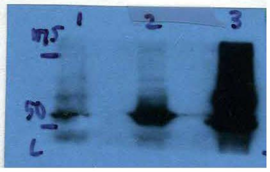

BioRad ChemDoc

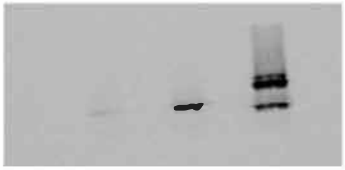

The same blot was exposed
to the film and scanned by ChemDoc.

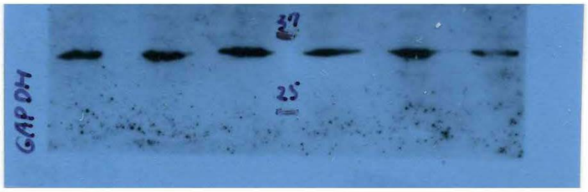

x x x 1 2 3

Image from ChemDoc was used for GFP western blot
and lanes 1,2 ,3 from X-ray film were used for GAPDH western blot.

Image of X-ray film was obtained by scanning each film.
