# Supplementary material for: Selective killing of homologous recombination-deficient cancer cell lines by inhibitors of the RPA:RAD52 protein-protein interaction
Source: PLoS One. 2021 Mar 30;16(3):e0248941. doi: 10.1371/journal.pone.0248941 (PMC8009417; doi:10.1371/journal.pone.0248941)
Supplement: Attachment — Submitted filename: Response to Review_1_29_2021.docx [file pone.0248941.s007.docx]

The reviewers’ comments are summarized below and followed by our response in purple italics. Places in the manuscript that were modified were highlighted in gray.

**Reviewer #1:** This is a very well written manuscript with novel and exciting data on using FDA approved drugs for targeted therapy of BRCA-1/2 cancers by specifically target the RPA:RAD52 protein interaction.

Comments:

1. Figures need to be of higher quality/resolution/contrast

*The resolution of all figures has been improved.*

2. Identification of the axes in some figure need to be more clear for example figure 1 y axes "% inhibition of " what?) While it is in the text and the legend it will be more clear if it was on the axes itself

*The y-axis for Figure 1B was revised to indicate what is being inhibited (RPA:RAD52 complex).*

3. Most of the supplemental data should be actually in the main manuscript that S2-6 figures as the some claims and conclusions in the manuscript cannot stand without the data in these figures. The data in the manuscript should stand by itself even without the supporting material.

*Although the data in the supplement support the manuscript conclusions, we consider these data as preliminary and this is an active area of research in the lab. Therefore, we believe the supplementary section is the appropriate place so they do not detract from the focus of the manuscript.*

4. Indicate significance in each figure where applied (use * or similar marking)

*The data in Figures 2, 3, and 4 were re-evaluated using ANOVA honesty testing as described in the edited method section of the manuscript and an asterisk sign was indicated where significance was found.*

5. Unpaired student t test is not an adequate statistical tool for some the data that have more than three variables authors need to do ANOVA with honesty testing.

*ANOVA with honesty testing was used to re-evaluate the statistical significance of the data as described in the revised method section.*

6. Minor spelling and grammar issues that can be easily fixed

*The manuscript was edited for any spelling or grammar issues*
Overall all the manuscript have excellent experimental design, adequate controls and pass the scientific rigor and it would be outstanding with these minor suggestion an comments.

**Reviewer #2:** In current manuscript, authors identified three FDA-approved drugs mitoxantrone, doxorubicin and quinacrine as the inhibitors of the RPA:RAD PPI by high throughput screening from chemical libraries. They demonstrated that these drugs could induce synthetic lethality in BRCA deficient cancer cell lines in vitro. Overall this is a preliminary study. Some points need to be addressed.

1. To address that RAD52 PPI inhibitors induce synthetic lethality in HR-deficient cancer cell lines, in Fig2-4, it should show the RAD52 expression in the tested cell lines before and after treatment. RAD52 is DNA Repair Protein. It also would be better to have the evidence that these drugs induced DNA damage in the tested cell lines, such as markers γH2AX.

*Thank you, this was an excellent idea and the resulting data proved to be very informative. All cell lines were grown and cells were harvested without treatment and treated with doses of mitoxantrone near their EC50 values. The level of DNA damage induced (γ-H2AX) and RAD52 expression was measured by western analysis as detailed in the method section. The western data were measured in triplicate, normalized with actin and graphed (average and standard deviation) with significance calculated using ANOVA: Single factor in Excel. These graphs were included as a new figure (Fig. 5) and so the figures after that were renumbered. Individual data measurements were included in the supplementary section using the lane representation (S2 Fig.). Western data were measured using a Protein Simple Peggy Sue instrument and analyzed using the associated Compass for Simple Western software.*

2. Fig3A, X axis missed concentration label “uM.

*Corrected.*

3. Fig 5 (now Fig 6), is it possible to show western blot original images (parts and FACS image?

*Western images are in Fig S6. The detailed FACS analysis from our core facility was added to Fig S6.*

4. FigS3A, B, it was not clear which cell line was treated. Can authors label them?

*Corrected.*

5. Fig S4, Western blot didn’t show loading control although authors claimed there were GAPDH controls in the experiments. (Not sure it was the image loading problem, it seemed Fig S4 missed left panel?)

*We did not describe that we used GAPDH as a loading control. We used p70 subunit of RPA to normalize the expression of SceI. All information is given in the legend.*

6. Labels in figure make people confuse. Fig S5, what does mean “1,2,3,4,5,6”? Was drug dose? FigS4, S5, was “c” control? Scel expressed in Fig S4”c” but not in Fig S5 “c”. What was the difference of the “C”?

*We have modified the labeling of Fig. S4 so the meaning is clearer. Now, in both figures, C stands for no SceI control. The 1,2,3,4,5,6 in Fig. S5 are simply lane numbers as described in the figure legend. We have revised the figure legends for both Figs. S4 and S5 for clarity.*
