# Supplementary material for: Selective killing of homologous recombination-deficient cancer cell lines by inhibitors of the RPA:RAD52 protein-protein interaction
Source: PLoS One. 2021 Mar 30;16(3):e0248941. doi: 10.1371/journal.pone.0248941 (PMC8009417; doi:10.1371/journal.pone.0248941)
Supplement: Attachment — Submitted filename: PLOS_One_response to review 2.docx [file pone.0248941.s008.docx]

EPPLEY INSTITUTE FOR CANCER RESEARCH

UNMC Fred & Pamela Buffett Cancer Center

National Cancer Institute Designated

Clinical Cancer Center

NEBRASKA’S HEALTH SCIENCE CENTER

March 1, 2020

Dr. Alvaro Galli

Academic Editor

PLOS ONE

Dear Dr. Galli,

We have implemented the suggestions from the reviewer by moving three figures from the supplement into the manuscript for the data that was directly cited in the text. There is a total of 9 figures in the main manuscript. The figures that remain in the supplement are only cited in the figure legends as they contain the original data that is summarized in graphs in the main figure. Some methods were moved from figure legends (previously in the supplement) to the methods section. These changes are highlighted in grey in the “Revised manuscript with Track Changes” document.

Concerning the resolution of the figures, we have uploaded high resolution figures. We are not sure why the figures the reviewer sees appear to be low resolution.

Thank you for your consideration.

Sincerely,

Gloria Borgstahl, Ph.D. and Tadayoshi Bessho, Ph.D.

Eppley Institute for Research in Cancer and Allied diseases

University of Nebraska Medical Center

UNMC Fred & Pamela Buffett Cancer

Omaha, NE 68198-6805, USA

Email: gborgstahl@unmc.edu; tbessho@unmc.edu
